# Europe Faces Multiple Arboviral Threats in 2025

**DOI:** 10.3390/v17121642

**Published:** 2025-12-18

**Authors:** Yannick Simonin

**Affiliations:** Pathogenesis and Control of Chronic and Emerging Infections, Montpellier University, INSERM, CHU Montpellier, 34000 Montpellier, France; yannick.simonin@umontpellier.fr

**Keywords:** arboviruses, chikungunya virus, dengue virus, West Nile virus, emergence, Europe

## Abstract

The year 2025 likely marks a turning point in both the perception and the reality of mosquito-borne arboviral diseases in Europe. While chikungunya and dengue viruses have long been regarded as tropical illnesses confined to intertropical regions, West Nile virus has circulated for decades in temperate areas, including southern Europe. Nevertheless, all three mosquito-borne viruses are now increasingly established across the European continent. This evolution reflects a profound transformation of the European epidemiological landscape, where arboviral diseases are increasingly emerging as endemic and seasonal threats. This shift concerns not only the scale but also the dynamics of transmission, with the appearance of newly affected regions, an earlier onset of the transmission season, and a broader diversity of arboviruses involved. Europe is thus entering a new phase in which longer, wider, and more intense transmission of vector-borne diseases is likely to become the new norm requiring strengthened preparedness.

## 1. An Intensification of Mosquito-Borne Arboviral Circulation

First identified in Europe in 1962, in southern France, West Nile virus (WNV) is an emerging orthoflavivirus that continues to spread across the European continent [1,2]. It is maintained in transmission cycles involving ornithophilic mosquitoes and resident or migratory birds acting as amplifying hosts, while humans and horses are epidemiological dead-end hosts [1]. In humans, infection is most often asymptomatic, with only 20 to 30% of infected individuals developing clinical signs. The mild form, known as West Nile fever, manifests as moderate fever, headache, muscle and joint pain, fatigue, and sometimes skin rash. In a minority of cases (approximately 1% of infections), the infection can progress to severe neurological forms such as meningitis, encephalitis or acute flaccid paralysis [3]. Over the following decades, WNV gradually established itself in multiple European regions, with increasing reports from southern, central, and eastern countries. The largest European WNV epidemic occurred in 2018, with 2083 human cases and 181 deaths recorded, marking an important milestone in the spread of this virus in Europe [2].

According to the epidemiological bulletins of the European Centre for Disease Prevention and Control (ECDC) published in November 2025, 14 European countries reported 1,096 locally acquired cases of WNV infection, including 95 deaths [4]. Cases were detected across parts of Eastern, Central, and Southern Europe, including Romania, Spain, Hungary, Croatia, Albania, Germany, North Macedonia, Bulgaria, Kosovo, and Turkey. Italy reported the highest number of cases in 2025, followed by Greece, Serbia, and France, accounting for nearly three-quarters of infections (773 confirmed cases), the majority of which were neuroinvasive forms. The most affected regions remain in central and southern Italy, but the virus has also spread further north, notably in Lombardy, Piedmont and Veneto. The observed case fatality among neuroinvasive cases is around 15%, a figure consistent with previous seasons, but highlighting the potential severity of WNV, which is by far the arboviral disease responsible for the most deaths in Europe each year [5]. Greece and Serbia were also among the most affected countries, with dozens of locally acquired cases reported in localized outbreaks, indicating intense and persistent virus circulation. In Greece, provinces around Athens and the northern part of the country have experienced recurrent seasonal outbreaks, often associated with significant human and equine mortality. In Serbia, the virus circulates mainly in the Belgrade region and along the Danube, where regular outbreaks have been documented for several years, confirming the endemicity of WNV in the Mediterranean basin and the Balkans [4]. France, until now less affected than some of its southern European neighbors, reported 59 locally acquired WNV cases during the 2025 season, along with its first three recorded deaths [6]. Cases were mainly concentrated in the southeast, but notably, around twenty cases were identified in Île-de-France, in the north-central region, and even within Paris proper. This represents an unprecedented geographic expansion, extending far beyond the typical areas of virus circulation around the Mediterranean. This development aligns with trends already observed in previous seasons, with sporadic outbreaks in the southwest (notably in Nouvelle-Aquitaine, where several human and equine cases had been recorded as early as 2022–2023) [7]. The northward spread, culminating in 2025 with the detection of locally acquired cases in the Paris region, marks a new stage in the virus’s expansion across French territory.

In 2025, dengue virus (DENV, *Orthoflavivirus denguei*) continued to circulate in temperate regions of Europe, as observed in previous years, although the incidence of locally acquired cases remained lower than in the preceding two years [8]. This viral infection most often manifests as sudden fever, severe headaches, joint and muscle pain, skin rash, as well as nausea and vomiting. In some cases, DENV can induce severe manifestations, such as hemorrhagic dengue or dengue shock syndrome, which may result in significant bleeding, circulatory failure, and, without appropriate care, death [9]. DENV is the most widely circulating arboviral disease in the world, with 14.1 million cases reported in 2024 [10]. In France, 29 locally acquired cases, distributed across 12 outbreaks, were recorded mainly in the south, but also in previously unaffected regions such as Grand Est, Nouvelle-Aquitaine, and Bourgogne-Franche-Comté [6,11]. Over the same period, 1132 imported dengue cases were identified. In Italy, authorities reported four locally acquired cases, two cases were also identified in Portugal. Although these numbers are modest compared with the historical peak of 304 locally acquired cases recorded in 2024 (including 213 in Italy, 83 in France and 8 in Spain) or the 130 cases in 2023, they reflect the virus’s ongoing establishment across the European continent [12]. Regarding serotype distribution, DENV-1 and DENV-2 were the predominant serotypes detected in autochthonous cases in France and Italy, consistent with previous years. No public evidence of DENV-3 or DENV-4 circulation has been reported in continental Europe in 2025, although limited serotyping data mean that their circulation cannot be entirely excluded. This serotype dynamic is critical, as co-circulation of multiple serotypes could significantly increase the risk of severe disease and future epidemic potential [13].

After several relatively quiet years in Europe, chikungunya virus (CHIKV, *Alphavirus chikungunya*), a member of the family *Togaviridae*, genus *Alphavirus*, emerged in 2025 as by far the most prominent arbovirus in terms of incidence and public health impact, with unprecedented levels of circulation. This virus can cause fever, headaches, skin rashes, and intense joint pain, sometimes chronic and severe [14]. Moreover, in elderly people, immunocompromised individuals, and newborns, serious systemic or neurological complications can occur, which are sometimes fatal [14]. Its resurgence in Europe this year was largely concentrated in France which experienced an unprecedented geographical expansion of transmission, both in both the scale and geographic range of transmission. Between May and October, nearly 800 locally acquired cases reported across 80 identified clusters while 1,073 imported cases were detected [6]. Although virus introductions clearly contributed to seeding these events, the large number of autochthonous cases and the recurrence of multi-case clusters demonstrate that sustained local transmission was the main driver of the chikungunya circulation in France in 2025. The first autochthonous case was detected in Strasbourg, located in the northeastern part of the country at a latitude historically considered unsuitable for sustained arboviral transmission. This observation highlights the growing capacity of *Aedes albopictus* populations, the mosquito species responsible for CHIKV transmission in Europe, to establish and remain competent vectors even in temperate regions, likely facilitated by progressive climatic warming and vector adaptation. In addition to the Strasbourg cluster, multiple foci of local transmission were documented in southeastern and southwestern regions of France. Several urban areas experienced repeated introductions followed by local spread, underscoring the combination of high human mobility, dense vector populations, and climatic conditions conducive to transmission during the extended summer season of 2025. Italy, which experienced a major CHIKV outbreak in 2017 with over 400 reported cases, reported more than 380 locally acquired cases in 2025, mainly in the provinces of Latina and Frosinone, south of Rome [11]. 

**Figure 1:**
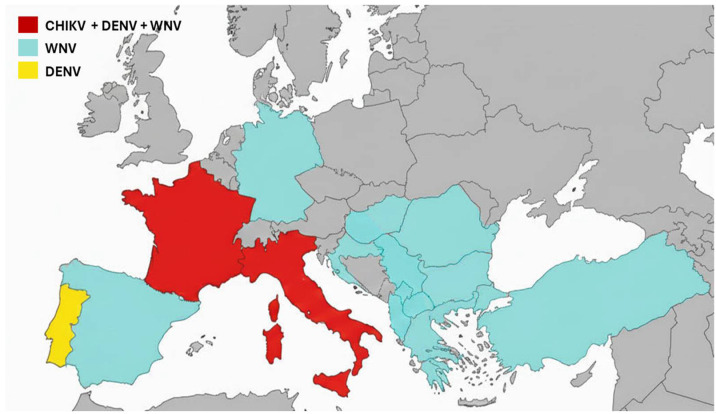
Autochthonous cases of CHIKV, DENV, and WNV in Europe in 2025. WNV cases were reported in Albania, Bulgaria, Croatia, France, Germany, Greece, Hungary, Italy, Kosovo, North Macedonia, Romania, Serbia, Spain, and Turkey. DENV cases were reported in France, Italy, and Portugal. CHIKV cases were reported in France and Italy.

Thus, the year 2025 likely marks an epidemiological turning point: for the first time, continental Europe simultaneously experienced circulation of three major mosquito-borne arboviruses, WNV, CHIKV, and DENV (Figure 1). This situation demonstrates that arboviruses are no longer merely a sporadic phenomenon in Europe, but a threat capable of establishing sustainable transmission in temperate regions of the continent. Mainland France has experienced an exceptional year of arboviral circulation, with nearly 900 locally acquired cases recorded, far exceeding the previous record of 122 cases reported just last year. Along with Italy, it is one of the two most affected European countries [4,6]. In 2025, France ranked as the most impacted country for both CHIKV and DENV cases and was among the most impacted countries for WNV infections. This particular dynamic in France can be explained by several factors. First, its proximity to overseas territories, notably La Réunion and Mayotte, two French overseas departments located in the Indian Ocean that experienced the largest chikungunya epidemic in over twenty years [15]. Regular air travel between these overseas territories and mainland France, as well as with other regions experiencing high viral circulation, has facilitated the introduction of the virus to the continent. The long-established presence of *Aedes albopictus*, the vector of DENV and CHIKV in southern France, and its expansion into three-quarters of metropolitan departments in recent years, has enabled the emergence of locally acquired cases across much of the territory. The diversity of affected regions, from the southern Mediterranean to eastern continental areas, underscores the expanding risk. This development calls for a revision of local vulnerability maps for arboviral diseases.

## 2. Culex Pipiens and Aedes Albopictus: Two Vectors at the Heart of Arboviral Spread in Europe

As noted above, a key driver of this arboviral expansion is *Aedes albopictus*, commonly known as the tiger mosquito and originally native to Southeast Asia. This species has progressively colonized Europe over the past two [16,17,18] In 2025, it is established in 16 European countries and 369 regions, compared with 114 regions in 2015 [19]. Its ability to adapt to urban environments, survive moderate winters through egg diapause, and reproduce in small volumes of standing water makes it a vector particularly well-suited to establishment and persistence in Europe. It can transmit arboviruses such as Zika virus (ZIKV), DENV, and CHIKV via an anthropophilic mode of transmission, relying on strictly human cycles without animal reservoirs (human–mosquito–human) [16]. Another key vector involved in arboviral transmission in Europe is *Culex pipiens*, a native mosquito widely distributed across the continent. This species is notably capable of transmitting WNV and the closely related Usutu virus (USUV, *Orthoflavivirus usutuense*), through an enzootic cycle between birds and mosquitoes [1]. Well adapted to urban and peri-urban environments, it feeds preferentially on birds but can occasionally bite humans or horses, resulting in accidental infections in these hosts, which act as epidemiological dead-ends. Thus, the combination of an urban, anthropophilic vector (*Aedes albopictus*) and an ornithophilic, widespread vector (*Culex pipiens*) illustrâtes the ecological complementarity of mosquitoes driving the current arboviral dynamics in Europe. 

While *Aedes albopictus* remains the dominant invasive vector in continental Europe, *Aedes aegypti* has recently re-emerged in parts of the European region, notably in Cyprus and Madeira [20,21]. This species is a more efficient vector for both DENV and CHIKV, owing to its strong anthropophily, high vector competence, and ability to thrive in densely populated urban environments [22]. Although *Aedes aegypti* has not yet established itself in continental Europe, its re-emergence in these territories represents a significant increase in epidemic potential. Moreover, beyond their current known vector associations, several arboviruses exhibit a remarkable capacity to adapt to new arthropod species, a phenomenon that can profoundly influence their geographic spread and epidemic potential. Such adaptive events have been well documented for CHIKV, in which single amino-acid substitutions in the E1 or E2 glycoproteins have increased viral fitness in *Aedes albopictus*, enabling efficient transmission in regions initially considered unsuitable [23,24]. Similar host–switching abilities have been described for ZIKV, highlighting the evolutionary plasticity of many arboviruses when exposed to novel vector populations [25]. This capacity for rapid adaptation underscores the need for continuous genomic and entomological monitoring, particularly in temperate areas where vector species are expanding.

## 3. Other Arboviral Threats Across Europe

Several additional mosquito- and tick-borne viruses represent potential threats for future emergence in Europe. Orthoflaviviruses such as ZIKV, yellow fever virus (YFV), and Japanese encephalitis virus (JEV) illustrate the capacity of *Aedes* and *Culex* vectors to sustain explosive outbreaks under suitable ecological conditions. The resurgence of ZIKV circulation in Brazil in 2025 highlight the potential risk of introduction through viremic travellers, particularly given the established presence of *Aedes albopictus* in large parts of Europe [26]. Moreover, several tick-borne arboviruses already circulate actively within Europe. Tick-borne encephalitis virus (TBEV) causes substantial human cases across central and eastern European countries [27]. The recent identification of Alongshan virus (ALSV) and other members of the Jingmen tick virus group in multiple European countries illustrates the continent’s vulnerability to previously unrecognised tick-associated viruses with zoonotic potential [28]. In addition Crimean–Congo haemorrhagic fever (CCHF) transmitted by *Hyalomma ticks,* continue to circulate in eastern and southeastern Europe, with recurrent human outbreaks reported in Bulgaria, Turkey, and the Balkans [29]. Moreover USUV has established endemic transmission in several European countries over the past decade, primarily affecting wild birds but occasionally causing neuroinvasive disease in humans, underscoring the ongoing risk posed by orthoflaviviruses in temperate regions [30]. Other, less well-known arboviral threats exist, and we must remain vigilant and prepared for the possible emergence of one or more new arboviruses in Europe.

## 4. Convergence of Factors Favoring Emergence and Re-Emergence, with Climate Change as a Primary Driver

The continuous rise in arboviral cases in Europe results from the convergence of several additive factors. Climate change is altering distribution ranges of arthropod vectors and lengthening transmission seasons. Consequently, the mosquito-borne virus transmission season now starts earlier and ends later. Rising average temperatures also promote viral replication within the vector and accelerate its blood-feeding cycle, thereby increasing the probability of viral transmission [31]. Most climate models predict a northward expansion of transmission across the continent, accompanied by increasingly prolonged transmission windows [32,33]. Traveler flows, notably tourism and migration, facilitate virus introduction via viremic travelers. Furthermore, rapid, often poorly planned urbanization creates environments favorable to mosquito proliferation, providing multiple larval breeding sites in urban and peri-urban areas. This phenomenon can be further amplified by poorly regulated urban greening strategies, which generate shelters for mosquitoes and promote larval habitats. Moreover, most European countries lack historical experience in managing arboviral diseases. Surveillance systems are often fragmented, healthcare professionals are insufficiently trained to recognize clinical manifestations of arboviral infections, and vector control policies are highly heterogeneous, sometimes even within the same region. This situation limits the capacity for rapid and coordinated responses.

The climatic conditions of 2025 were particularly favorable for arboviral transmission, with one of the warmest years on record. The year was marked by early heat episodes (late May–early June) and daily temperatures frequently exceeding 30 °C, with nights often remaining above 20 °C, while the preceding winter was milder than usual [34]. In addition, precipitation patterns included intense rainfall followed by dry periods, favoring the accumulation of standing water suitable for mosquito oviposition. Altogether, these conditions enabled prolonged vector activity and promoted the emergence of local epidemic foci, with transmission peaks observed from May through the end of September.

Another important driver of arbovirus expansion is biodiversity loss. Reduced predator populations and simplified ecosystems favor arthropod vectors proliferation by eliminating natural regulatory mechanisms. This ecological imbalance, combined with urbanization, creates optimal conditions for vector expansion and arbovirus transmission [35,36]. Moreover globalisation accelerates the spread of invasive vectors. International trade, particularly in used tires and ornamental plants, has facilitated the introduction of *Aedes* species from endemic regions to Europe. Combined with increased air travel, these pathways enable rapid dissemination of both vectors and viruses [37]. Increased freight volume, accelerated delivery timelines, and expanding commercial routes all amplify the probability that vector species are transported from endemic regions to Europe. 

## 5. Major Challenges for European Public Health

Europe is entering a phase in which arboviral diseases are becoming seasonal endemic threats. This evolution poses several major challenges. Entomological and epidemiological surveillance remain insufficient in many European countries, not only because systems are unevenly implemented, but also due to several structural limitations. Case definitions and reporting criteria differ between countries, complicating direct comparisons of incidence and hindering timely interpretation of epidemiological trends [38,39]. Diagnostic laboratory capacities remain highly heterogeneous across Europe. Some countries rely on only a few reference facilities, whereas others lack the capability to perform confirmatory molecular or serological testing, leading to diagnostic delays and substantial underreporting. In addition, surveillance of arboviral diseases still relies largely on passive clinical reporting, a system in which mild or atypical cases, particularly common for DENV or WNV, are frequently missed. Vector surveillance programs are equally heterogeneous, varying widely in frequency, methodology, and the entomological indicators monitored. While some countries perform routine mosquito trapping and viral testing, others operate only seasonal or reactive surveillance, generating gaps in early warning capacity. Collectively, these limitations hinder coordinated risk assessment and delay the implementation of effective vector-control measures. With case numbers still relatively low, healthcare professionals are not always adequately trained to recognize the polymorphic symptoms of chikungunya or other arboviruses such as WNV. Cross-border coordination is also suboptimal, with vector control policies varying widely between countries and data sharing remaining limited.

A more comprehensive approach is therefore essential to better anticipate epidemic waves, coordinate interventions, and harmonize responses. Effective preparation requires the development of strategic priorities at the European level. Integrated surveillance, combining entomological, climatic, and clinical data, needs to be strengthened. Tools such as real-time vector mapping and climate modeling could help anticipate the highest-risk periods and enable more targeted surveillance measures.

Diagnosis of arboviral diseases represents another major challenge. Clinical presentations are often nonspecific, multiple virus strains and lineages with varying virulence may co-circulate, and the limited availability of rapid, sufficiently specific and sensitive tests complicates early detection. Cross-reactivity observed in many serological, and even molecular, tests further hampers diagnostic reliability. It is therefore crucial to strengthen the diagnostic capacities of European laboratories, including university hospitals, to continue developing multiplex tools suitable for European contexts and to promote access to testing in high-risk areas, including in primary care. In this context, structured laboratory networks and a degree of centralization of highly specialized capacities are essential to ensure diagnostic quality and efficiency [39,40] The recent designation of the European Union Reference Laboratory for Public Health on Vector-borne Viral Pathogens provides a framework to harmonize protocols, distribute reference materials, coordinate external quality assessments and support national expert laboratories for confirmatory testing and training

Vaccine development must also be accelerated. Two chikungunya vaccines, IXCHIQ (Valneva) and Vimkunya (Bavarian Nordic), have recently been approved in Europe [41,42,43]. Ensuring their availability in high-risk areas and for vulnerable populations, including the elderly, immunocompromised individuals, and healthcare workers, is essential to limit the impact of future outbreaks. Regarding DENV, several vaccine candidates are under evaluation or already available in certain countries. However, their variable efficacy depending on serotype and prior immunity necessitates a cautious and targeted deployment strategy [44,45]. For WNV, no human vaccine is currently available, unlike equine vaccines, which have proven effective. Research in this field must therefore be supported to develop preventive solutions tailored to the most at-risk populations. In addition, the development of antiviral therapies should be pursued to provide treatment options for infected individuals, particularly those at higher risk of severe disease.

Improved training of healthcare professionals constitutes another key pillar of the response. Arboviral diseases should be more fully integrated into continuing medical education programs, and clinical guidelines adapted to new European contexts should be widely disseminated. Community engagement is also crucial. Public awareness campaigns on preventive measures, such as eliminating larval habitats and using personal protective strategies, should be strengthened, particularly in urban and peri-urban areas, which are most exposed to arboviral risk.

A One Health approach, integrating human, animal, and environmental health sectors, is essential for arbovirus monitoring, particularly for WNV, given the diversity and still incomplete knowledge of the avian reservoir species involved in its transmission. This requires integrated strategies that combine entomological, veterinary, and human surveillance activities, as well as enhanced collaboration between human and animal health sectors to enable rapid risk identification. Integrated One Health surveillance systems involving mosquitoes and birds across several European countries have already demonstrated their usefulness for the early detection of WNV circulation, with Italy providing a particularly illustrative example of how coordinated, multi-sectoral monitoring can be effectively implemented. Such systems must be integrated with USUV surveillance, as this virus co-circulates with WNV in both space and time. Their eco-epidemiology relies on complex interactions between *Culex* vectors, diverse avian reservoirs, and incidental mammalian hosts, and may complicate diagnosis due to serological cross-reactivity [1]. Likewise, horses act as highly sensitive sentinel hosts for WNV, providing a critical interface between veterinary and human health surveillance. Strengthening these animal-health components, together with robust local and national integrated surveillance programs, is essential for the timely and effective detection of future outbreaks and for improving Europe’s preparedness to manage potential epidemic situations.

## 6. Conclusions

The year 2025 should not be seen as an anomaly in the epidemiological picture of mosquito-borne arboviral circulation in Europe but as a warning signal. Arboviral diseases are now clearly identified as seasonal threats in Europe and public health systems must adapt to this changing situation. It is time to shift from a reactive mindset to a sustained culture of preparedness, integrating arboviruses into public health plans, engaging citizens, and investing in research, surveillance, and vaccination. Only by implementing these measures can Europe face this new epidemiological reality, in which viruses once considered tropical are becoming recurrent seasonal threats.

## Data Availability

No new data were created or analyzed in this study.
